# Effects of a 10 vs. 20-Min Injury Prevention Program on Neuromuscular and Functional Performance in Adolescent Football Players

**DOI:** 10.3389/fphys.2020.578866

**Published:** 2020-10-15

**Authors:** Anna Lina Rahlf, Cornelius John, Daniel Hamacher, Astrid Zech

**Affiliations:** Department of Human Movement Science and Exercise Physiology, Institute of Sport Science, Friedrich Schiller University Jena, Jena, Germany

**Keywords:** sensorimotor control, physical performance, proprioception, youth football (soccer), injury prevention

## Abstract

**Background:**

Regular injury prevention training is not only effective in reducing sports injury rates, but also in improving neuromuscular and performance-related variables. However, it is currently unknown if this effect can be modified by varying the training dosage.

**Objective:**

To compare the effects of two injury prevention programmes with a different training duration on neuromuscular control and functional performance in adolescent football players.

**Methods:**

342 (15.4 ± 1.7 years) male football players from 18 teams were initially included. The teams were cluster-randomized into two intervention groups. Both groups performed an injury prevention program twice a week during one football season (10 months) using the same exercises but a different duration. One intervention group (INT10, *n* = 175) performed the program for 10 min, while the other intervention group (INT20, *n* = 167) for 20 min. At the beginning and end of the season, balance control (Balance Error Scoring System = BESS), jump performance (Squat Jump, Countermovement Jump) and flexibility (Sit and Reach Test, ankle flexibility, hip flexibility) tests were performed. For the final analysis, nine teams with 104 players were considered.

**Results:**

Significant group by time interactions were found for the sit and reach test (*p* < 0.001) and ankle flexibility (*p* < 0.001) with higher improvements in the INT20 group. Improvements over the period of one season but no group differences were found for the BESS, Squat Jump and hip flexibility.

**Conclusion:**

Within a single training session, performing structured neuromuscular training with a longer duration is more effective than a shorter duration for improving lower extremity flexibility.

## Introduction

Football (soccer) requires a range of conditional and coordinative skills that must be quickly available under various technical and tactical conditions (Ali, 2011). Along with a good endurance performance, these skills require a high degree of neuromuscular control and functional abilities from players. The improvement of neuromuscular performance is described as a key element in the prevention of injuries in football (Impellizzeri et al., 2013). The training of these neuromuscular skills requires both general and specific exercises in agility, balance, plyometric skills, power, stability and strength (Myer et al., 2011). According to this, most injury prevention programmes contain both general and specific exercises (Mugele et al., 2018) and are often described as multimodal injury prevention programmes. The use of such multimodal approaches appears to be not only effective in reducing injuries (Rössler et al., 2014; Soomro et al., 2016), but also in improving neuromuscular performance (Faude et al., 2017) in adolescent athletes. A recent meta-analysis by Faude et al. (2017) found small to high effects on balance, stability, leg power, leg strength, sprint, and sport-specific performance variables. While this emphasizes the overall effectiveness of neuromuscular training there are still considerable differences between the reported effects of the included studies (Faude et al., 2017). One possible reason is speculated to be the high variety in the training dosage, including factors such as duration, frequency and volume between existing studies. Accordingly, although the effectiveness of neuromuscular training is well established, the dose-response-relationship is widely unknown. In a previous study, the influence of different session durations of neuromuscular training was investigated and showed similar effects on lower extremity injuries in youth football players performing shorter (10 min) compared to longer (20 min) session durations (Rahlf and Zech, 2020). These results are supported by the systematic review of Steib et al. (2017), who found an association between the training dose and the effect sizes of neuromuscular training studies focusing on injury prevention in adolescent athletes. The most beneficial effects were found for programmes in which session durations lasted 10 to 15 min or longer, the training was performed at least twice weekly and the overall program volume included between 20–60 sessions within a 6 months period (Steib et al., 2017). There is a consensus in the literature that the reduced injuries associated with improved neuromuscular control and functional performance (Soligard et al., 2008; Alentorn-Geli et al., 2009; Impellizzeri et al., 2013). It is therefore only logical that effective injury prevention programmes target neuromuscular parameters. However, to the best of our knowledge, no study has experimentally analyzed the influence of the training dose on improvements in neuromuscular control and functional performance variables.

Therefore, the aim of this study was to compare the effects of two injury prevention programmes on neuromuscular and functional performance, using the same exercises with different session durations. Based on the current state of research, the improvement of neuromuscular and functional performance is considered relevant for injury prevention effects. The few studies considering dosage parameters of neuromuscular training demonstrated positive effects in injury prevention with short bouts. Accordingly, we hypothesized that both groups will improve in neuromuscular and functional performance, irrespective of session duration. The results help to provide practical recommendations for more tailored injury prevention programmes.

## Materials and Methods

**FIGURE 1:**
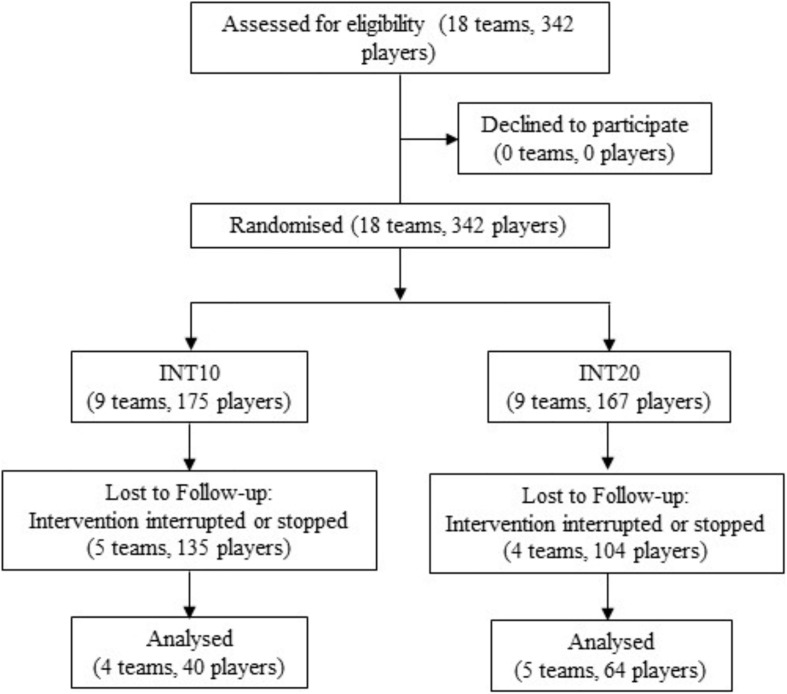
Flow diagram of team and player participation through the study process.

A cluster-randomized controlled trial was conducted between August 2016 and June 2017 (Figure 1). Due to teams typically practicing collectively, the concealed random cluster allocation was made for the entire team. Group allocation was performed considering age and level of play, whereby teams of the same playing level and similar age were matched and randomly allocated to one of the intervention groups by pulling a concealed envelope. An allocation to the INT10 group implied that the players had to perform the neuromuscular training for 10 min twice a week and INT20 for 20 min twice a week.

The recruitment took place in local football clubs and youth football academies in North and East Germany. Inclusion criteria for participation were male football players from Under 14 (U14) to Under 19 (U19), who trained at least twice a week in addition to matches. Players with acute injuries including loss of training and match were excluded from pre- and post-test. Initially, a telephone enquiry was made to the coaches, followed by a personal visit to give more detailed information and to provide a written study description to the coaches and athletes. After meeting the criteria of eligibility, participants or their legal representatives gave their written informed consent. Due to the voluntary participation, the players could withdraw from the study without giving any reasons. All measurements were performed according to the general ethical guidelines based on the Declaration of Helsinki, and ethically approved by the local ethics committee (No. FSV 17/02).

### Procedure

Players were tested at the beginning and at the end of the intervention period (one season, 10 months). A battery of standardized functional performance test was used, and the order of tests was randomized. In order to address the several components of the multimodal training program, the battery consisted of balance, jump performance and flexibility tests. Prior to the assessment, stature and body mass of each player were determined. According to existing literature the Balance Error Scoring System (BESS), Squat Jump (SJ), and Countermovement Jump (CMJ) the Thomas Test (TT), Sit and Reach Test (SR), and Weight-bearing Lunge Test (WBLT) were performed (Kaufman et al., 1999; Soderman et al., 2001; Dallinga et al., 2012; Robles-Palazón et al., 2016; Ayala et al., 2017; Faude et al., 2017; Trecroci et al., 2018).

#### Balance Test

Balance was measured using the BESS test. The BESS is a valid and reliable measure to assess balance control in young and healthy athletes (Docherty et al., 2006; Clark et al., 2010; Bell et al., 2011). The players stood barefoot with hands on the iliac crests and eyes closed in six conditions: double-leg, single-leg and tandem stance on a firm and foam surface. During each condition the players had to stand as motionless as possible for 20 s. The kicking-leg was considered as dominant limb (Steffen et al., 2013), and was chosen for the single-leg stance and as the rear leg during the tandem stance. During each test condition, the numbers of the following errors were counted: lifting hands off iliac crests, opening eyes, stepping, stumbling, falling, moving the hip more than 30°, lifting the forefoot or heel, remaining out of the testing position for more than 5 s (Bell et al., 2011). In order to ensure an excellent reliability, the measurements were filmed and rated by the same tester. For statistical purposes, the total number of errors across all six conditions was analyzed.

#### Flexibility Tests

Hip flexibility was measured with the original Thomas Test (Peeler and Anderson, 2007). The test was chosen because it doesn’t require many materials and it’s more practicable for the measurements in the field. The player was in the supine position holding the uninvolved limb bent with both hands close to the chest, to control the lumbar lordosis. A standard goniometer was used to measure the hip angle of the relaxed and passive test leg. When it has been ensured that no evasive movements and only gravity affected the leg, the tester placed the goniometer on the lateral aspect of the greater trochanter to measure the hip angle. The horizontal position of the test leg was defined as zero position.

The Sit and Reach Test is a well evaluated and widely used tool to assess hamstring and lower back flexibility (Ayala et al., 2012; Mayorga-Vega et al., 2014). The players sat upright on the floor with flexed hips and extended knees, and both feet touching each other. The soles of the feet were placed against the edge of the assessment box. On top of the box, a measuring scale was placed to identify the reach distance in cm, positioning the feet at the level of zero. During the test, the players reached their extended arms with one hand placed on the other and with palms down as far as possible along the scale without bending knees or lifting heels from the box. The maximum reach distance of three trials was used for data analysis.

The Weight-bearing Lunge Test (WBLT) was used to assess the dorsiflexion range of the ankle. For the test a good to excellent reliability is reported (Powden et al., 2015). The players stood barefoot in a lunge position, facing a wall, with one foot placed 5 cm apart from the wall on a measuring tape, placed on the floor. They were challenged to touch the wall with their knee without lifting the heel. In case the players were able to complete the task, they moved the foot backwards along the tape until the maximum reach distance without lifting the heel. The maximum reach distance between the wall to the big toe was measured in cm. This method has previously been used in other studies (Bennell et al., 1998; Hoch and McKeon, 2011; Konor et al., 2012; Hartley et al., 2018).

#### Vertical Jump Tests

Vertical jump tests were used to analyze the athletes’ explosive power performance which is a fundamental skill in football (Kotzamanidis et al., 2005). Players performed a SJ and CMJ. During the SJ, the players moved in a deep squat position with 90° knee flexion angle for ∼3 s before jumping upward as high as possible. The CMJ was performed from an upright standing position going downwards to a self-selected bending position of the knees followed by maximum jump height. Both jumps were performed with shoes and hands on the hips. Each player completed three repetitions per jump condition and the highest jump was used for further analyses (Kotzamanidis et al., 2005). The jump height was measured by wearable inertial measurement units (IMUs) (Sensor DX3.1, Humotion, Muenster, Germany) secured by a strap on the back of the players. Previously research has shown that the use of IMUs is a valid device to determine jump performance (Picerno et al., 2011; MacDonald et al., 2017).

### Intervention

The 11+ is a standardized injury prevention program in football (Bizzini and Dvorak, 2015), and was used as a substitute warm-up intervention to the usual warm-up routine. The program was performed in its original (INT20) and a modified (INT10) version twice a week during one football season (10 months). Prior to the season, each coach was verbally instructed and practically trained in the 11+ program by a physiotherapist and the primary investigator. Additionally, the coaches received detailed information and illustrations using the official manual and poster. The original 11+ warm-up program is separated in three categories including 15 exercises: (1) running including flexibility; (2) strength, plyometrics, balance, stability; running, cutting, jumping (Bizzini et al., 2011). While the INT20 conducted the usual 11+ program for about 20 min twice weekly the INT10 group exercised twice weekly for only 10 min with the modified version of the 11+ program. The shorter time was realized by reducing the time or number of repetitions in each exercise. The number and type of exercises remained similar to the INT20 group. During the intervention period, the research team visited every football team twice to supervise and answer questions of the coaches and players while performing the injury prevention program.

### Statistical Analysis

The statistical analyses were performed using the Statistical Package for Social Science, version 25 (SPSS Inc., Chicago, Illinois, United States). A *T*-test was used to examine the differences between pre and post measures between groups. Data are shown as mean and standard deviation (SD). To address the main aim regarding group by time effects, each outcome measure (static balance, vertical jump performance, flexibility of the lower extremity) was analyzed using multilevel linear mixed models (random intercept models) to account for the nested data structure (teams, players and legs of the players). Group and time as well as the interaction effect group × time were modeled as fixed effects. Furthermore, the confounders BMI, age, injured and tested leg were included as fixed effects to consider potential bias. The results were calculated in estimated means and illustrated in graphs including 95% confidence interval (95% CI).

In all cases, the statistical significance was determined by an alpha level of 0.05.

## Results

Of the initially included 342 players, *n* = 175 players were allocated to the INT10 group and *n* = 167 to the INT20. Due to drop-out rate (INT10: 56%; INT20: 45%) during the season, four teams in the INT10 group (40 players) and five teams in the INT20 group (64 players) with complete pre and post test data were considered for analysis (Figure 1). Nevertheless, no relevant differences were found in the preseason baseline anthropometric data considering the initial population and the included population (Table 1). The distributions of playing level, age groups and training sessions between the intervention groups are also shown in Table 1. In the INT10 group the modified injury prevention program was performed in 59 (SD 21, range 28–75) of a total of 126 training sessions (47%) with an adherence rate of 79%. The INT20 group completed 60 sessions (SD 11, range 53–77) of the 11+ program out of 129 training sessions (47%) with an adherence rate of 80%. Taking into account the number of minutes of the 11+ training, the INT10 group completed 593 min on average and the INT20 group 1208 min on average (Table 1).

**TABLE 1:** Baseline demographics and team characteristics of the sample separated in pre and post-test (only analyzed sample).

	Pre			Post		
	INT10 (*n* = 175)	INT20 (*n* = 167)	*p*-value	INT10 (*n* = 40)	INT20 (*n* = 64)	*p*-value
Age (years)	15.2 ± 1.7	15.5 ± 1.6	0.446	16.4 ± 1.7	16.7 ± 1.2	0.313
Height (Cunningham et al., 2007)	172.8 ± 0.1	173.7 ± 0.9	0.161	176.7 ± 0.1	176.1 ± 0.1	0.679
Weight (Putukian et al., 2015)	63.0 ± 11.8	62.6 ± 11.3	0.777	67.1 ± 10.5	66 ± 8.3	0.559
Body mass index (kg/m^2^)	20.9 ± 2.6	20.6 ± 2.4	0.919	21.4 ± 2.5	21.2 ± 2.1	0.700
Level of play (number of teams)						
Elite	1	–		–	–	
Sub-elite	2	5		1	3	
Recreational	6	4		3	2	
Age group (number of teams)						
U14	1	1		2	1	
U15	2	2		1	1	
U16	1	1		0	1	
U17	2	2				
U19	3	3		1	2	
Training sessions (mean and SD)						
Units per season	–	–		126 (43)	172 (58)	
11+ units	–	–		59 (21)	60 (11)	
11+ min	–	–		593 (212)	1208 (217)	

### Effects of Confounders on Functional Performance Tests

Regarding confounders, age significantly influenced (*p* < 0.001) jump performance and the BMI influenced hamstrings/lower back (*p* = 0.028) and ankle flexibility (*p* = 0.015). Furthermore, leg side showed a significant influence on the outcomes of the Thomas test (*p* < 0.001). No significant effect was found for the confounder injured or not injured.

### Effects of Session Duration on Functional Performance Tests

**FIGURE 2:**
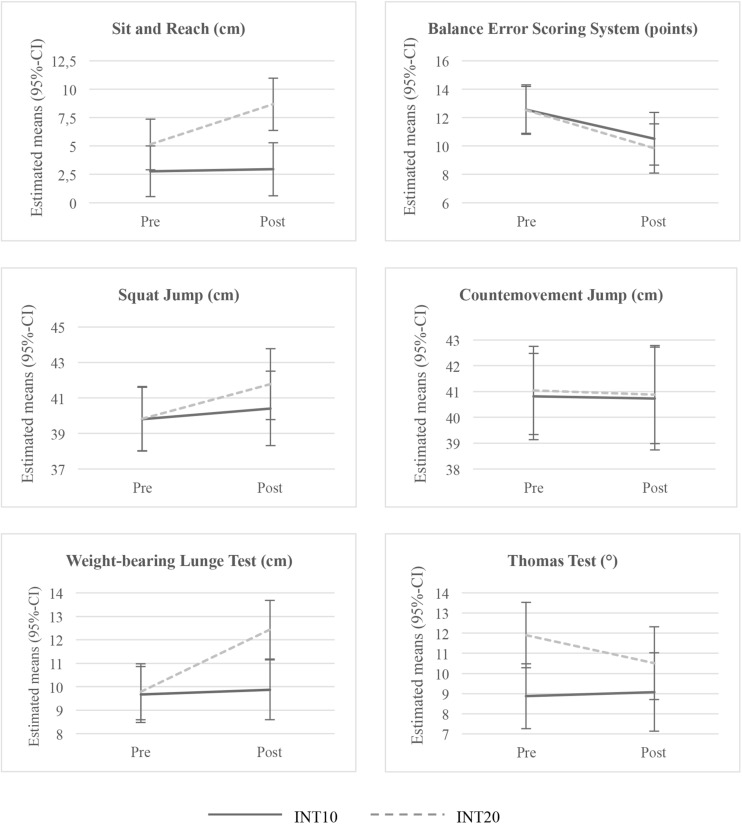
Estimated means and 95%-CI after adjustment for confounders of the two intervention groups for Sit and Reach, BESS, Squat Jump, Countermovement Jump, WBLT and Thomas Test.

Comparing the two session durations, significant group by time interactions were found for the Sit and Reach (*p* < 0.001) and the WBLT (*p* < 0.001) (Table 2). The INT20 increased significantly compared to the INT10 (b: −5.73, SE 1.57, *p* < 0.001) and over the time (b: −3.55, SE 0.84, *p* < 0.001) in the Sit and Reach performance (Table 2 and Figure 2). The same result was observed for the WBLT. The INT20 group significantly increased their ankle flexibility (WBLT) from pre to post test (b: −2.65, SE 0.38, *p* < 0.001) as well as compared to the INT10 group at post-test (b: −2.56, SE 0.85, *p* = 0.006) (Table 2 and Figure 2). No significant interactions were found for the BESS, SJ and CMJ (Table 2). Further time effects in the INT20 group were found for the BESS (b: 2.71, SE 0.88, *p* = 0.002) and Thomas Test (b: 1.39, SE 0.69, *p* = 0.044) (Table 2 and Figure 2).

**TABLE 2:** Group wise comparison of INT10 and INT20 expressed as estimater (b) and 95% Confidence interval (95% CI) considering pre and post measurement including confounder analysis for age, BMI, tested leg and injured or not injured.

Fixed effects		Sit and reach (Cunningham et al., 2007)	BESS (sum score)	Squat jump (Cunningham et al., 2007)	Countermovement Jump (Cunningham et al., 2007)	WBLT (Cunningham et al., 2007)	Thomas test (°)
Intercept	b	−6.05	4.20	**15.34**	**16.30**	**13.50**	6.55
	95% CI	[−18.65,−6.54]	[−4.64,13.05]	[6.42,24.25]	[8.04,24.55]	[7.40,19.60]	[−2.05,15.15]
	*p*	0.338	0.337	0.001	<0.001	<0.001	0.132
	*t* (df)	−0.97 (44.15)	0.98 (25.23)	3.50 (32.95)	4.06 (25.67)	4.43 (61.17)	1.54 (43.23)
Group (INT10 = 0) (reference: INT20 = 1)	b	−**5.73**	0.68	−1.37	−0.15	−**2.56**	−1.43
	95% CI	[−8.97,−2.49]	[−1.85,3.22]	[−4.28,1.54]	[−2.91,2.61]	[−4.32,−0.80]	[−4.04,1.19]
	*p*	0.001	0.584	0.351	0.914	0.006	0.274
	*t* (df)	−3.64 (26.16)	0.55 (27.52)	−0.94 (66.45)	−0.12 (64.36)	−3.02 (21.76)	−1.11 (33.03)
Time (pre = 0) (reference: post = 1)	*b*	−**3.55**	**2.71**	−1.95	0.16	−**2.65**	**1.39**
	95% CI	[−5.20,−1.90]	[0.97,4.44]	[−4.34,0.43]	[−2.18,2.50]	[−3.28,−2.03]	[0.04,2.74]
	*p*	<0.001	0.002	0.108	0.896	<0.001	0.044
	*t* (df)	−4.25 (142.91)	3.08 (172.12)	−1.62 (168.97)	0.13 (241.44)	−8.36 (209.93)	2.03 (220.52)
Group*Time (INT20*pre)	*b*	**3.37**	−0.66	1.34	−0.07	**2.45**	−1.60
	95% CI	[1.72,5.02]	[−2.53,1.20]	[−1.30,3.97]	[−2.68,2.53]	[1.67,3.22]	[−3.48,0.27]
	*p*	<0.001	0.482	0.318	0.955	<0.001	0.094
	*t* (df)	4.05 (111.54)	−0.71 (135.45)	1.00 (128.44)	−0.06 (201.66)	6.23 (123.78)	−1.69 (163.20)
Age (15.46 yrs)	*b*	0.35	0.08	**1.58**	**1.62**	0.18	0.12
	95% CI	[−0.43,1.12]	[−0.47,0.63]	[1.02,2.14]	[1.10,2.13]	[−0.19,0.55]	[−0.41,0.66]
	*p*	0.372	0.770	<0.001	<0.001	0.332	0.643
	*t* (df)	0.90 (58.44)	0.30 (32.89)	5.71 (40.66)	6.36 (32.38)	0.98 (76.47)	0.47 (51.03)
BMI (20.64 points)	*b*	**0.44**	0.24	0.09	0.03	−**0.19**	0.14
	95% CI	[0.05,0.83]	[−0.02,0.50]	[−0.18,0.36]	[−0.23,0.29]	[−0.35,−0.04]	[−0.08,0.35]
	*p*	0.028	0.066	0.498	0.804	0.015	0.218
	*t* (df)	2.21 (342.82)	1.84 (283.09)	0.68 (304.10)	0.25 (323.06)	−2.45 (333.91)	1.23 (315.53)
Tested leg (left leg = 0) (reference: right leg = 1)	*b*	–	–	–	–	−0.14	−**1.28**
	95% CI	–	–	–	–	[−0.32,0.04]	[−1.63,−0.94]
	*p*	–	–	–	–	0.138	<0.001
	*t* (df)	–	–	–	–	−1.49 (436.65)	−7.37 (433.33)
Injuries (not injured = 0) (reference: injured = 1)	*b*	0.62	−1.26	0.28	−1.99	0.33	−0.29
	95% CI	[−0.99,2.22]	[−2.98,0.46]	[−2.08,2.65]	[−4.29,0.30]	[−0.17,0.84]	[−1.26,0.69]
	*p*	0.449	0.149	0.813	0.089	0.194	0.567
	*t* (df)	0.76 (118.156)	−1.45 (190.13)	0.24 (231.81)	−1.71 (316.69)	1.30 (568.17)	−0.57 (593.64)

*Alpha-level ≤ 0,05.*

## Discussion

The main finding of this study was a positive influence on lower extremity flexibility in young football players performing the longer version of injury pervention training sessions compared to a shorter version over one season (10 months). In detail, beneficial effects were found for hamstring and ankle flexibility after the 20 min program when compared to the 10 min program. Although some studies reported both decreased flexibility and hyper mobility as injury risk factors (Dallinga et al., 2012), only few studies investigated the influence of injury prevention programmes on flexibility (Robles-Palazón et al., 2016; Ayala et al., 2017). Accordingly, the present results provide novel and important information about the effects of these exercises included in the programmes. Although no significant effects were found for the other neuromuscular control and functional performance variables, there were considerable changes in the estimated means. The INT20 group improved in the BESS, SJ, and Thomas Test while the INT 10 group showed improvements only in the BESS score. Thus, the results show speculative initial indications that performing a 20 min injury prevention program during one football season may be more effective for improving functional performance than a 10 min training program. To the best of our knowledge, our study is the first to prospectively analyse the dose-response-relationship of a neuromuscular training program on neuromuscular and functional performance, as existing studies regarding the effectiveness of the 11+ were solely performed across the original duration of 20 min. In a previous randomized controlled trial, significant effects compared to controls were found for functional balance in female youth football players performing the 11+, 2–3 times a week for 4.5 months (Steffen et al., 2013) as well as for male professional players performing the program 3 times a week for 2 months (Daneshjoo et al., 2012). Adolescent male futsal players practising the program 2 times a week for 12 weeks, improved significantly in the vertical jump performance (Reis et al., 2013). The same was found for adolescent football players performing the 11+, 3 times a week for 9 weeks (da Costa Silva et al., 2015) but not in adult players (Impellizzeri et al., 2013). However, performing the program 3 times a week for only 4 weeks (12 sessions) did not change hip or ankle flexibility in young amateur players (Robles-Palazón et al., 2016; Ayala et al., 2017). In contrast, after 60 total sessions with 20 min of neuromuscular training in the present study, we found significantly improved ankle flexibility. In the review including meta-analysis by Faude et al. (2017), the researchers found higher training adaptions in neuromuscular and physical performance when performing the prevention program more than 23 training sessions. Taking existing evidence and the results of the current study into account, we conclude that the effectiveness of neuromuscular training interventions on neuromuscular control and/or performance-related outcomes depends on different dosage parameters. Intervention periods with a higher number of sessions of longer duration seem more effective than those with fewer sessions of shorter duration. Even if the difference of 10 vs. 20 min seems to be slight, at the end, the INT20 group performed twice as much neuromuscular training than the INT10 group (593 ± 212 vs. 1208 ± 217 min). Usually, the training dose is divided into different categories: session duration, training frequency, weekly training volume, intervention volume and intervention period (Steib et al., 2017). Due to the lack of studies, the influence of most of the single dosage components on the effectiveness of the neuromuscular training intervention on neuromuscular and functional performance remains speculative. However, higher effects seem to depend on a higher overall intervention volume (number of sessions together with a longer session duration). Reviews with focus on dose-response of balance training found the highest effect on balance-related outcomes, an important part of neuromuscular control, in session durations of 11–15 min, training frequencies 3–6 time per week and intervention period of 12 weeks (approx. 36–72 training sessions) (Zech et al., 2010; Lesinski et al., 2015). Regarding injury outcomes, contradictory results were found. In the review including meta-analysis by Sugimoto et al. (2014) they found the highest effects of a neuromuscular training program for prevention of anterior cruciate ligament (ACL) injuries in female young athletes in session durations longer than 20 min, at least two sessions and more than 30 min weekly. In a follow-up review including meta-regression, the authors identified the dosage of neuromuscular training as critical component and reported less ACL injuries the more frequent and longer neuromuscular training was performed (Sugimoto et al., 2016). The meta-analysis of Steib et al. (2017) on injury prevention effects in youth athletes found the highest effect for session durations with at least 10–15 min, a frequency of 2–3 times per week and a weekly training volume of 30–60 min. However, contrary to our findings, the intervention volume and period and the overall numbers of sessions did not influence the reduction of injury rates (Steib et al., 2017). In conclusion, it can be assumed that an overall volume of neuromuscular training must be achieved before an improvement in neuromuscular control or functional performance with the result of an effective injury prevention occur. Improved neuromuscular and functional performances are the results of increased strength, balance, flexibility, proprioception and endurance. Future work should therefore focus on separating the basic elements of neuromuscular performance to find minimum dosage parameter needed in order to improve neuromuscular performance. Further, the research should also focus on the time course of improvements in the single performance variables. Such investigations require that we separate the dosage parameters accurately to identify an adaption of neuromuscular performance based on training load and time.

### Limitations

Some limitations need to be addressed. To implement an intervention program in football practice requires a good compliance of the coaches. In the present study, the drop-out rate increased during the season to approximately 55%. A higher capacity to closely monitor compliance of the coaches and performance of the players could help to avoid high drop-out rates. However, the analyzed sample size is higher than in most comparable published studies (Daneshjoo et al., 2012; Impellizzeri et al., 2013; da Costa Silva et al., 2015; Robles-Palazón et al., 2016). Further, even if the intervention program was delivered identically to all coaches, consistent implementation was not ensured. Accordingly, coaches incorrectly controlling or prescribing the exercises may have impacted the physical performance results. In the present study, the neuromuscular and functional assessment was performed at the beginning and after 10 months of intervention. To closely evaluate the time-related improvements of physical performance, several measurements should be conducted during the intervention period. Since the test battery contained just a small range of neuromuscular and functional performance tests, further specific tests should also be used in future investigations. The present results relate entirely to young male football players and cannot be transferred to other populations. As this is a first study on the dose-response effects of an injury prevention program on neuromuscular and functional performance, the results must be interpreted with caution.

## Conclusion

The results of this study demonstrate that performing an injury prevention training for 20 min was more effective in improving ankle and hamstring flexibility than a shorter version of just 10 min in young male football players. Although improvements in static balance, vertical jump performance and hip flexibility were not significant, longer training sessions showed a larger influence on improved functional performance. These results may help to provide practical recommendations for more tailored injury prevention programmes in young football players. Due to the limitations and the lack of the ability to transfer the results to other populations, further research is needed.

## Data Availability Statement

The dataset for this article is not publicly available, however, requests to access the datasets should be directed to AR (anna.lina.rahlf@uni-jena.de).

## Ethics Statement

The studies involving human participants were reviewed and approved by the Ethical Commission of the Faculty of Social and behavioral Sciences, Friedrich Schiller University Jena, Jena, Germany. Written informed consent to participate in this study was provided by the participants’ legal guardian/next of kin.

## Author Contributions

AR designed and conceived the study, performed the data processing and the statistical analysis as well as wrote the manuscript draft. CJ assissted in the data collection. DH was involved in the statistical analysis. AZ supported the development of the initial idea and preparation of the study design. DH, CJ, and AZ critical revised the draft and read and approved the final version of the manuscript. All authors contributed to the article and approved the submitted version.

## Conflict of Interest

The authors declare that the research was conducted in the absence of any commercial or financial relationships that could be construed as a potential conflict of interest.
