# Supplementary material for: Effects of a 10 vs. 20-Min Injury Prevention Program on Neuromuscular and Functional Performance in Adolescent Football Players
Source: Front Physiol. 2020 Oct 15;11:578866. doi: 10.3389/fphys.2020.578866 (PMC7593709; doi:10.3389/fphys.2020.578866)
Supplement: Supplementary file 1 [file Data_Sheet_1.PDF]

Supplementary Material

Effects of a 10 vs. 20 minutes injury prevention programme on neuromuscular and functional performance in adolescent football players

Anna Lina Rahlf*, Cornelius John, Daniel Hamacher, Astrid Zech

*** Correspondence:** Anna Lina Rahlf (anna.lina.rahlf@uni-jena.de)

1 Differences of exercise repetitions for the original 11+ programme and the modified version.

**Exercise**	**Original repetitions** (INT20)	**Modified repetitions** (INT10)
**Part 1: Running exercises: including stretching and controlled partner contacts (variations in speed)**		
Running *straight ahead*	*2 laps*	*1 lap*
Running *hip out*	*2 laps*	*1 lap*
Running *hip in*	*2 laps*	*1 lap*
Running *circling partner*	*2 laps*	*1 lap*
Running *shoulder contact*	*2 laps*	*1 lap*
Running *quick forwards and backwards*	*2 laps*	*1 lap*
**Part 2: Strength, plyometrics, balance**		
The Bench<br>*Levels: 1: static, 2: alternate legs, 3: one leg lift and hold*	*Level 1: 3 sets, 30 sec.*<br>*Level 2: 3 sets, 60 sec.*<br>*Level 3: 3 sets, 30 sec.*	*Level 1: 2 sets, 20 sec.*<br>*Level 2: 2 sets, 45 sec.*<br>*Level 3: 2 sets, 20 sec.*
Sideways Bench<br>*Levels: 1: static, 2: raise and lower hip, 3: with leg lift*	*All levels and each side: 3 sets, 30 seconds*	*All levels and each side: 2 sets, 20 seconds*
Hamstrings<br>*Levels: 1: beginner, 2: intermediate, 3: advanced*	*Level 1: 5 rep.*<br>*Level 2: 10 rep.*<br>*Level 3: 15 rep.*	*Level 1: 3 rep.*<br>*Level 2: 7 rep.*<br>*Level 3: 12 rep.*
Single-leg stance<br>*Levels: 1: hold the ball, 2: throwing ball with partner, 3: test your partner*	*All levels and each side: 2 sets, 30 seconds*	*All levels and each side: 1 set, 30 seconds*
Squats<br>*Levels: 1: with toe raise, 2: walking lunges, 3: one-leg squats*	*Level 1: 2 sets, 30 sec.*<br>*Level 2: 2 sets, 10 each side*<br>*Level 3: 2 sets, 10 each side*	*Level 1: 1 set, 30 sec.*<br>*Level 2: 1 set, 10 each side*<br>*Level 3: 1 set, 10 each side*
Jumping<br>*Level 1: vertical jumps, 2: lateral jumps, 3: box jumps*	*All levels: 2 sets, 30 seconds*	*All levels: 1 set, 30 seconds*
**Part 3: Running exercises: including planting and cutting (high speed)**		
Running *across the pitch*	*2 laps*	*1 lap*
Running *bounding*	*2 laps*	*1 lap*
Running *plant and cut*	*2 laps*	*1 lap*
